# Supplementary material for: Plasma-Induced Oxidation Products of (–)-Epigallocatechin Gallate with Digestive Enzymes Inhibitory Effects
Source: Molecules. 2021 Sep 24;26(19):5799. doi: 10.3390/molecules26195799 (PMC8510274; doi:10.3390/molecules26195799)
Supplement: Supplementary file 1 [file molecules-26-05799-s001.zip › molecules-1349797-supplementary.pdf]

Supplementary Materials

Plasma-Induced Oxidation Products of (–)-Epigallocatechin Gallate with Digestive Enzymes Inhibitory Effects

Gyeong Han Jeong and Tae Hoon Kim *

Department of Food Science and Biotechnology, Daegu University, Gyeongsan 38453, Korea; jkh4598@hanmail.net

* Correspondence: skyey7@daegu.ac.kr; Tel.: +82-53-850-6533

Contents

Figure S1. Isolation procedure of plasma irradiated EGCG in aqueous solution.

Figure S2. HPLC chromatograms of isolated compounds **1-3**.

Figure S3. ^{1}H NMR spectrum of compound **1** in CD_3OD.

Figure S4. ^{13}C NMR spectrum of compound **1** in CD_3OD.

Figure S5. HSQC spectrum of compound **1** in CD_3OD.

Figure S6. HMBC spectrum of compound **1** in CD_3OD.

Figure S7. FABMS spectrum of compound **1**.

Figure S8. ^{1}H NMR spectrum of compound **2** in CD_3OD.

Figure S9. ^{13}C NMR spectrum of compound **2** in CD_3OD.

Figure S10. FABMS spectrum of compound **2**.

Figure S11. ^{1}H NMR spectrum of compound **3** in CD_3OD.

Figure S12. Calibration curve of EGCG and oxidation products **1-3**.

Figure S13. Chemical structures of isolated compounds **1-3**.

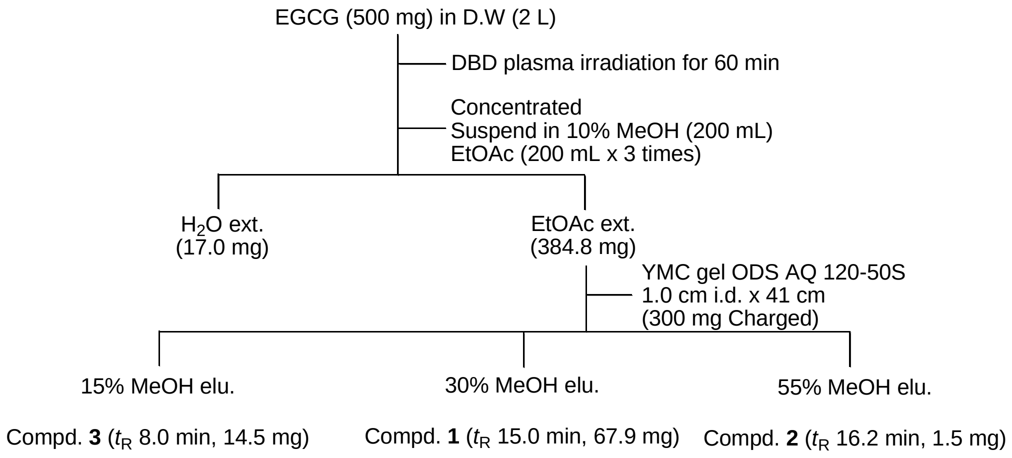

Figure S1. Isolation procedure of plasma irradiated EGCG in aqueous solution.

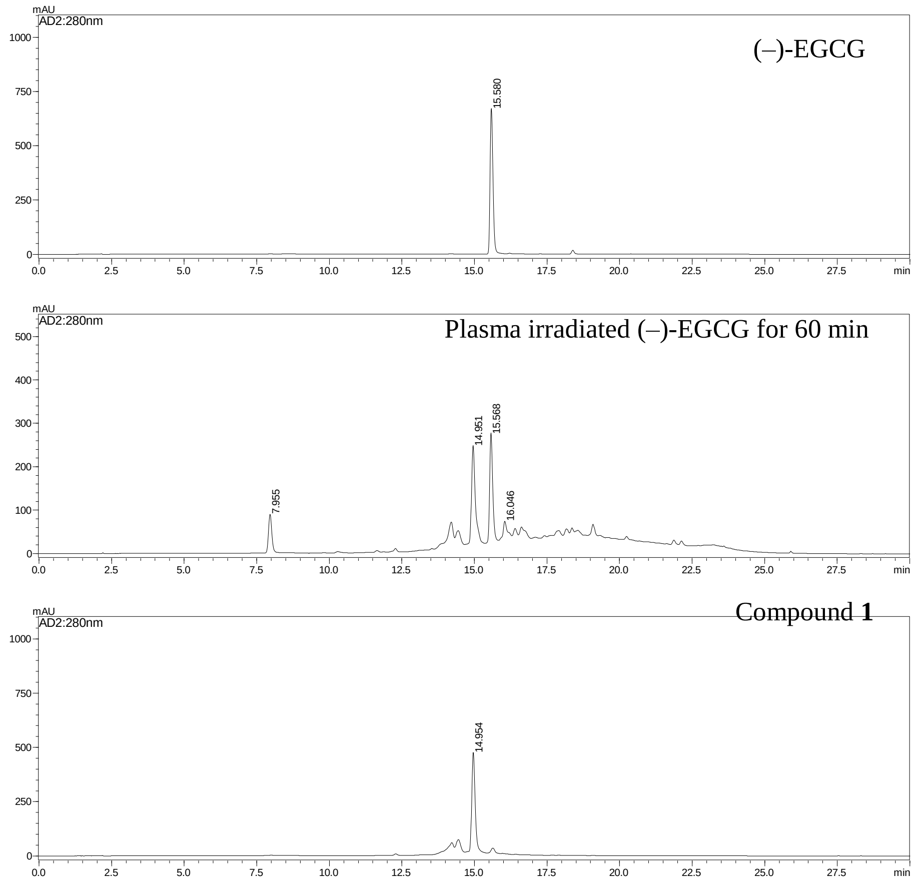

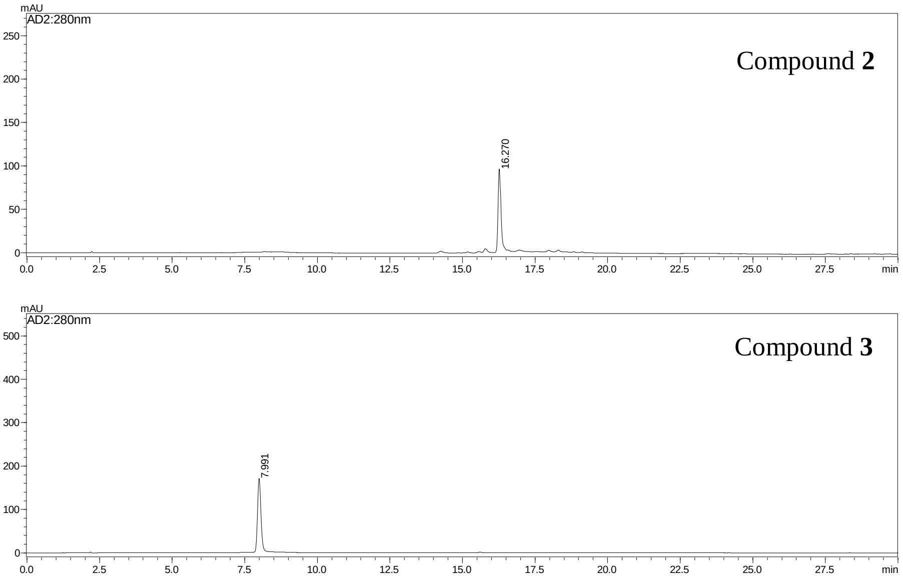

Figure S2. HPLC chromatograms of isolated compounds **1**-**3**.

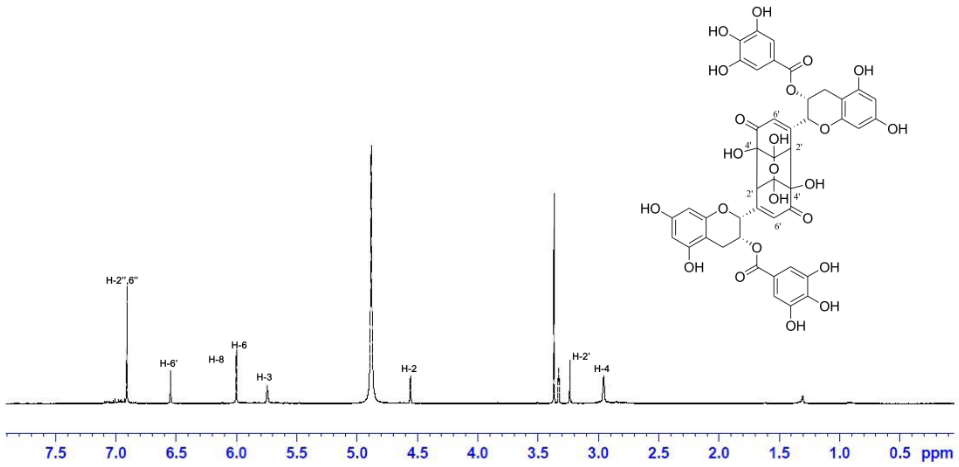

Figure S3. ^{1}H NMR spectrum of compound **1** in CD_3OD.

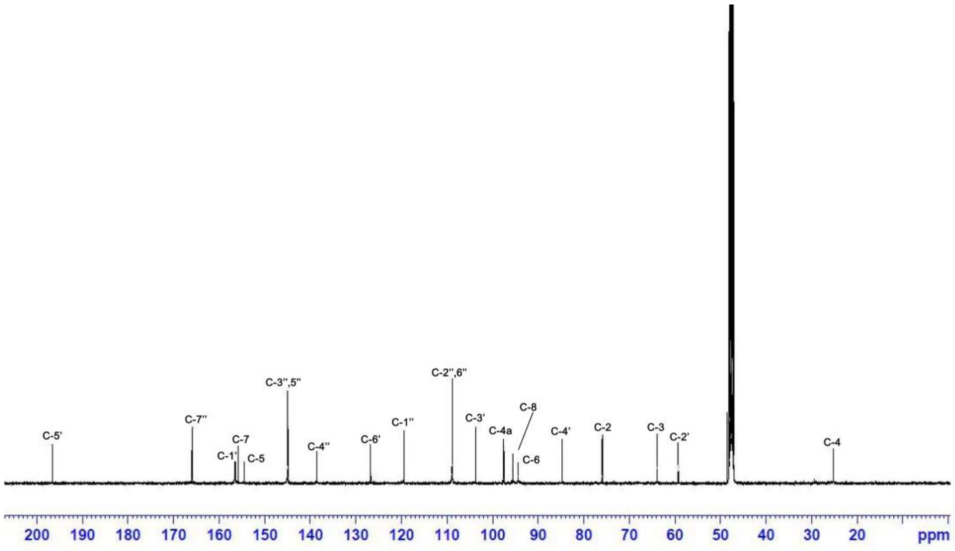

Figure S4. ^{13}C NMR spectrum of compound **1** in CD_3OD.

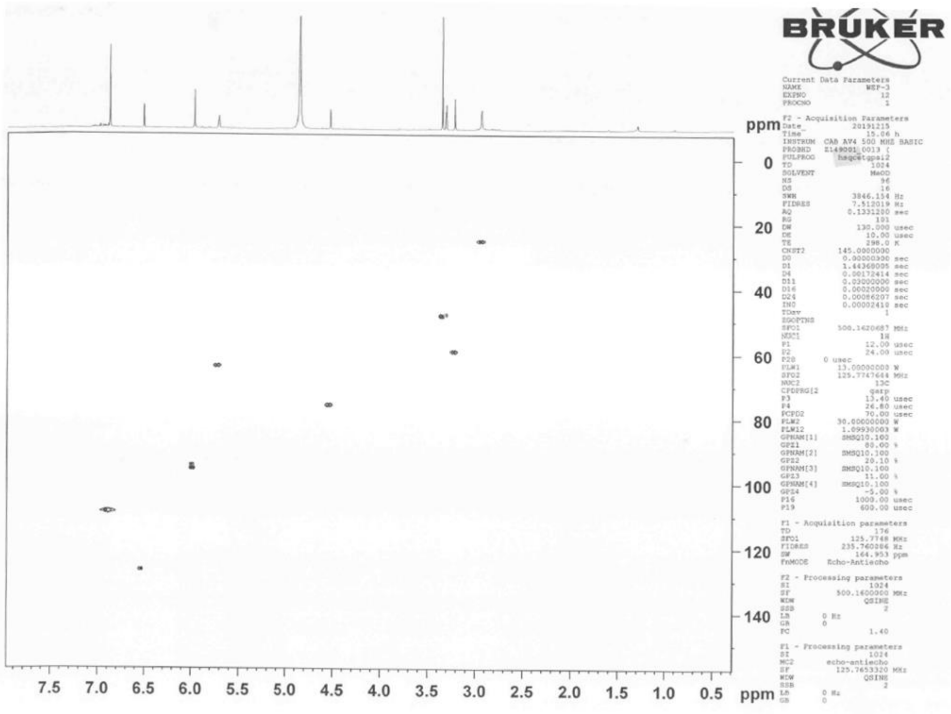

Figure S5. HSQC spectrum of compound **1** in CD_3OD.

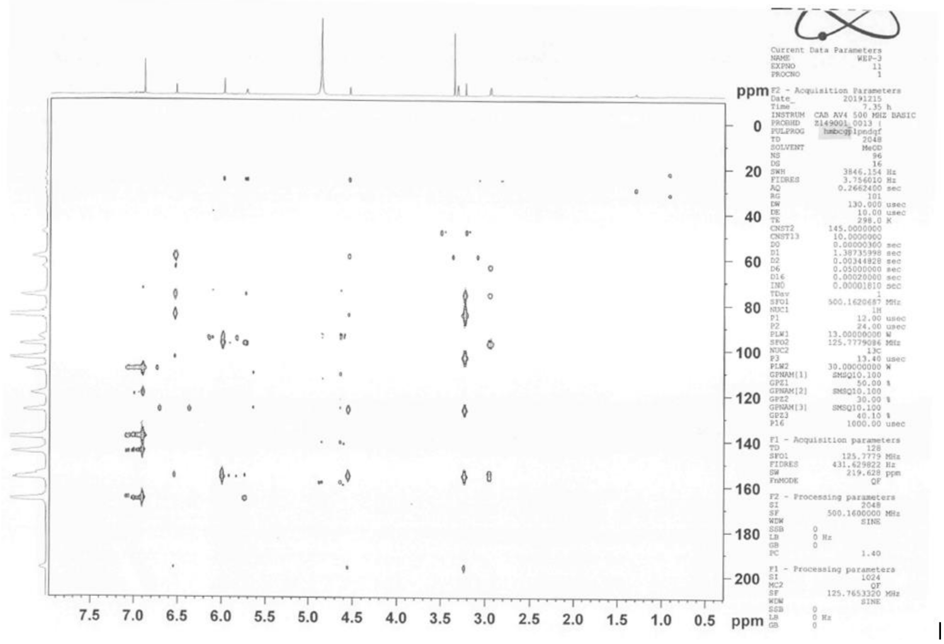

Figure S6. HMBC spectrum of compound **1** in CD_3OD.

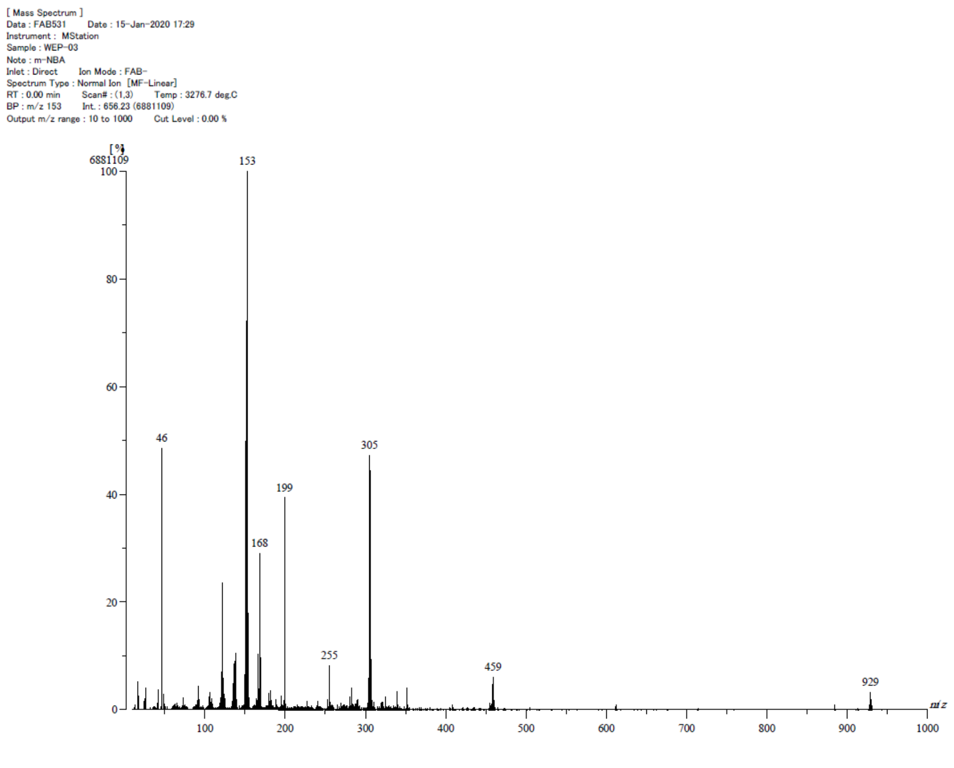

Figure S7. FABMS spectrum of compound **1**.

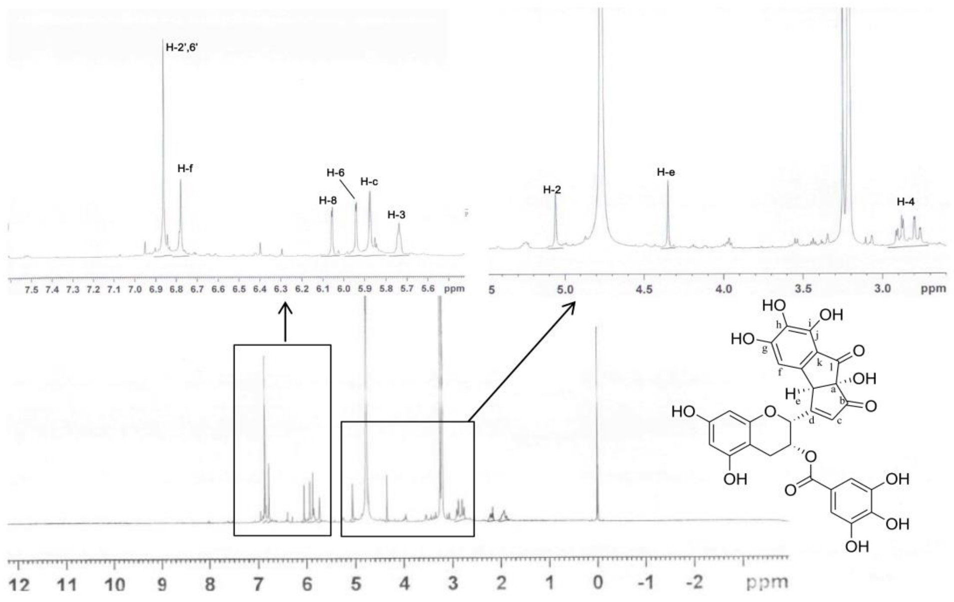

Figure S8. ^{1}H NMR spectrum of compound **2** in CD_3OD.

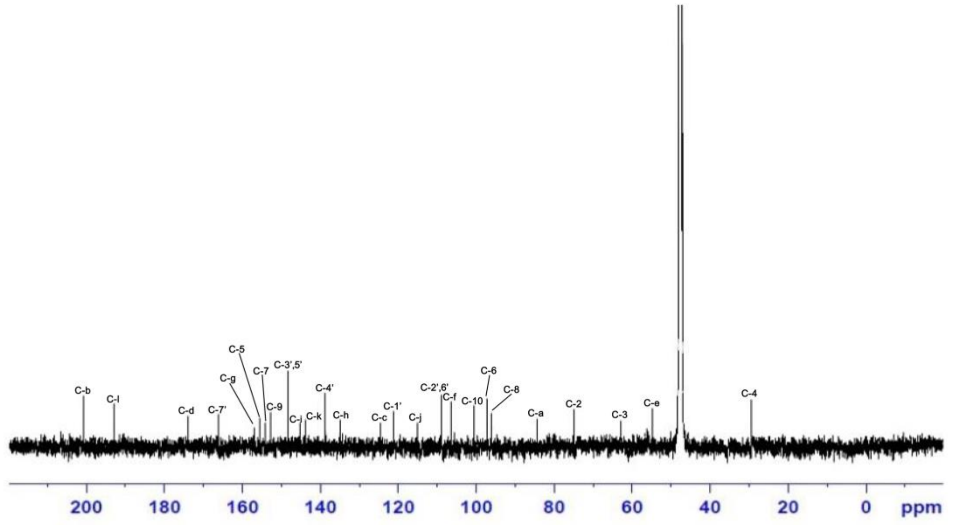

Figure S9. ^{13}C NMR spectrum of compound **2** in CD_3OD.

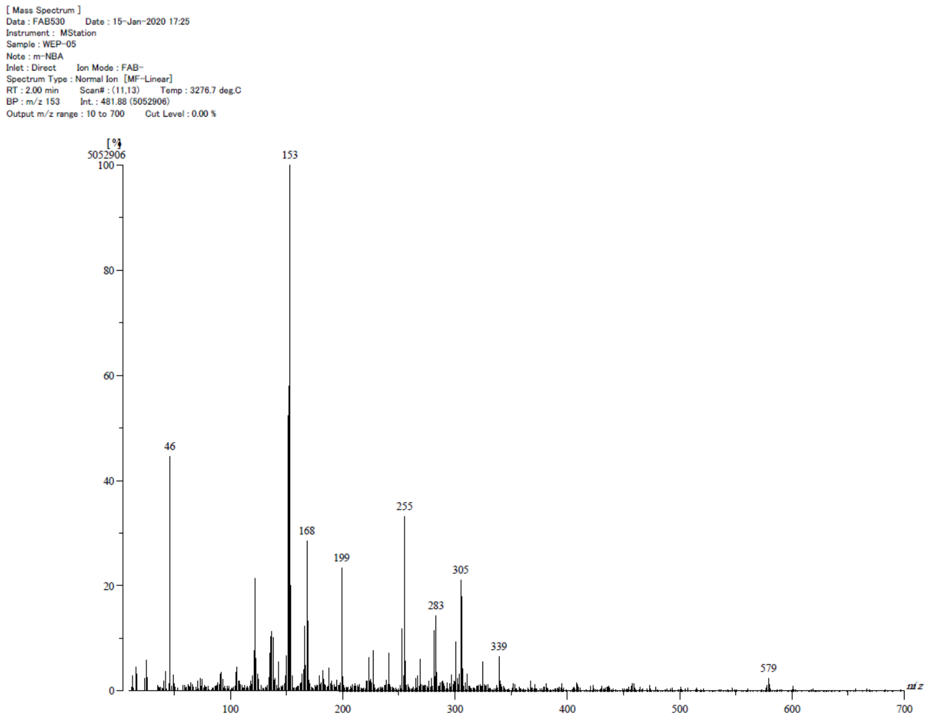

Figure S10. FABMS spectrum of compound **2**.

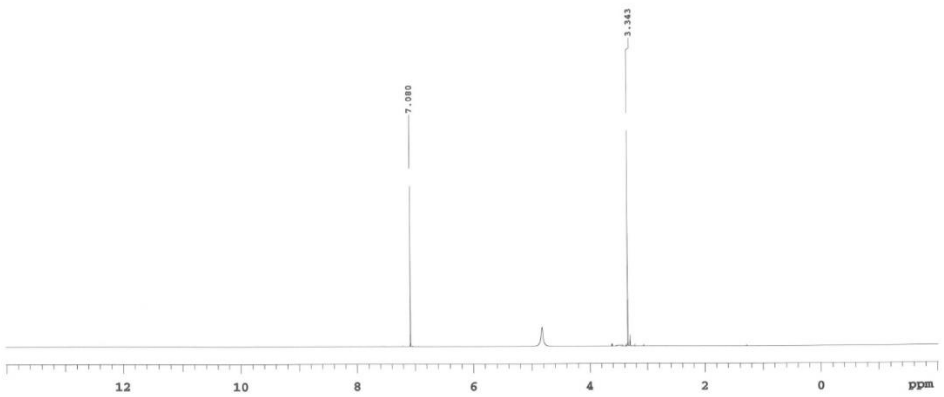

Figure S11. ^{1}H NMR spectrum of compound **3** in CD_3OD.

EGCG quinone dimer A (**1**): Brown amorphous powder, $[\alpha]^{25}_{D}$ -62.3 (*c* 0.1, MeOH), FABMS *m/z* 929 $[M–H]^-$, ^{1}H NMR (500 MHz, CD_3OD): δ 6.92 (4H, s, H-2'', 6''), 6.54 (2H, d, J = 1.0 Hz, H-6'), 6.03 (2H, d, J = 2.0 Hz, H-8), 5.99 (2H, d, J = 2.0 Hz, H-6), 5.68 (2H, br dd, J = 4.0, 1.0 Hz, H-3), 4.56 (2H, br s, H-2), 3.22 (2H, br s, H-2'), 2.95 (2H, m, H-4), 2.93 (2H, m, H-4), ^{13}C NMR (125 MHz, CD_3OD): δ 196.5 (C-5'), 165.9 (C-7''), 156.6 (C-7), 156.5 (C-9), 155.8 (C-1'), 154.4 (C-5), 144.9 (C-3'', 5''), 138.5 (C-4''), 126.7 (C-6'), 119.6 (C-1''), 108.9 (C-2'',

6''), 103.7 (C-3'), 97.7 (C-10), 95.6 (C-8), 94.4 (C-6), 84.7 (C-4'), 75.9 (C-2), 63.8 (C-3), 59.2 (C-2'), 25.2 (C-4).

Theacitrinin A (**2**): Brown amorphous powder, $[\alpha]^{25}_{D}$ +104.8 (*c* 0.1, MeOH), FABMS *m/z* 579 $[M–H]^{-}$, ^{1}H NMR (500 MHz, CD_3OD): δ 6.95 (2H, s, H-2', 6'), 6.71 (1H, s, H-f), 6.05 (1H, d, J = 2.0 Hz, H-8), 5.98 (1H, d, J = 2.0 Hz, H-6), 5.85 (1H, br s, H-c), 5.73 (1H, m, H-3), 5.23 (1H, br s, H-2), 4.35 (1H, s, H-e), 2.91 (1H, dd, J = 17.0, 4.0 Hz, H-4), 2.80 (1H, dd, J = 17.0, 1.0 Hz, H-4), ^{13}C NMR (125 MHz, CD_3OD): δ 200.1 (C-b), 193.9 (C-l), 174.0 (C-d), 166.0 (C-7'), 157.0 (C-g), 156.9 (C-5), 156.1 (C-7), 155.0 (C-9), 148.2 (C-3', 5'), 145.1 (C-i), 143.0 (C-k), 138.9 (C-4'), 134.0 (C-h), 125.0 (C-c), 122.0 (C-1'), 115.0 (C-j), 108.8 (C-2', 6'), 106.1 (C-f), 100.4 (C-10), 97.2 (C-6), 95.9 (C-8), 84.8 (C-a), 73.1 (C-2), 63.0 (C-3), 54.8 (C-e), 29.0 (C-4).

Gallic acid (**3**): White amorphous powder, ^{1}H NMR (500 MHz, CD_3OD): δ 7.08 (2H, s, H-2, 6).

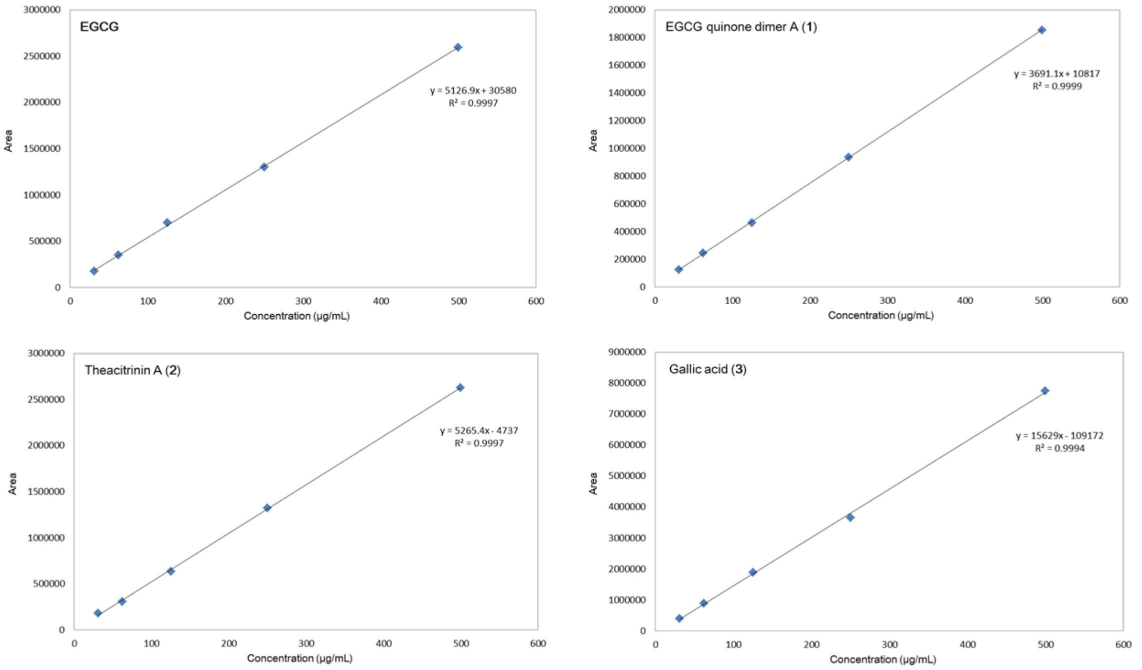

Compounds	t_R (min)	UV λ_{max} (nm)	$[M–H]^{-}$	Regression equation[a] (Y=aX+b, R^2)	Linear range (μg/mL)
(-)-EGCG	15.6	224, 280	457	Y = 5126.9X+30580, 0.9997	500-31.25
**1**	15.0	276	929	Y = 3691.1X+10817, 0.999	500-31.25
**2**	16.2	277, 345	579	Y = 5265.4X-4737, 0.9997	500-31.25
**3**	8.0	214, 268	169	Y = 15629X-109172, 0.9994	500-31.25

[a] Y = peak area and X = concentration.

Figure S12. Calibration curve of EGCG and oxidation products **1**-**3**.

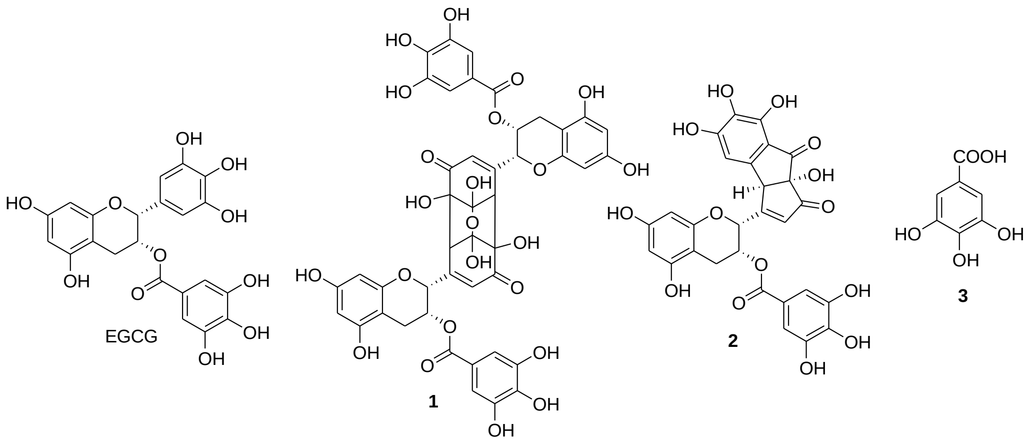

Figure S13. Chemical structures of isolated compounds **1**-**3**.
